# Therapeutic ecology of the fiber-microbiota-barrier axis in leukemia: resilience, immune recovery and pharmacomicrobiomics

**DOI:** 10.3389/fmicb.2026.1913406

**Published:** 2026-08-25

**Authors:** Ruiyu Xie, Xiaotong Jing

**Affiliations:** 1 Department of Gastroenterology, The Fourth Hospital of Hebei Medical University, Shijiazhuang, China; 2 Department of Hematology, The Second Hospital of Hebei Medical University, Hebei Key Laboratory of Hematology, Shijiazhuang, Hebei, China

**Keywords:** dietary fiber, gut microbiota, intestinal barrier, leukemia, microbial metabolites

## Abstract

Leukemia care is a clinical experiment in ecosystem stress. Malignant hematopoiesis, intensive chemotherapy, hematopoietic cell transplantation, neutropenia, mucosal injury, broad-spectrum antibiotics and nutritional interruption converge to deplete anaerobic fermentative capacity and favor pathobiont domination. The central gap is not whether dysbiosis occurs, but how loss of fiber-dependent microbial function becomes barrier failure, infection risk, immune dysregulation, treatment toxicity and altered drug exposure. We reviewed adult or non-pediatric full-text literature published from 13 May 2021 to 13 May 2026, after excluding pediatric-focused studies, and integrated foundational evidence on dietary fiber, microbial metabolites and cancer microbiome methodology. The literature supports a therapeutic ecology model in which fermentable substrate, anaerobic redundancy, short-chain fatty acids, indoles, bile-acid derivatives and amino-acid metabolites form a linked fiber-microbiota-barrier axis. When this axis is disrupted by antibiotics, mucositis, sterile or low-residue diets, parenteral nutrition and hospitalization, the ecosystem can enter alternative stable states marked by domination, resistome expansion and reduced metabolite output. Evidence is strongest in acute myeloid leukemia, acute leukemia chemotherapy and hematopoietic cell transplantation, but emerging studies in chronic lymphocytic leukemia, chronic myeloid leukemia, CAR T-cell therapy and pharmacomicrobiomics extend the framework beyond infection prevention. We therefore argue that microbiota-directed nutrition in leukemia should be designed as a timed, safety-bounded ecological intervention rather than as a generic supplement. Future trials should combine dietary quantification, antibiotic metrics, strain-level microbiome profiling, metabolomics, barrier biomarkers, drug-exposure readouts and patient-centered outcomes to test whether restoring the fiber-microbiota-barrier axis improves adult leukemia care.

## 1 Introduction

The gut microbiota is no longer a peripheral consideration in hematology. It is an organ-level ecological interface through which diet, antimicrobials, epithelial injury, mucosal immunity, systemic inflammation and antineoplastic therapy interact. Leukemia places this interface under extraordinary pressure. Patients may enter treatment with disease-associated inflammation, cytopenias, anorexia, altered metabolism and comorbid medication exposure. They then encounter chemotherapy, conditioning, transplantation, mucositis, neutropenia, antibacterial and antifungal prophylaxis, empiric broad-spectrum therapy and reduced enteral intake. In this environment, the microbial community can shift from a diverse anaerobic ecosystem to a simplified configuration dominated by facultative taxa with bloodstream infection or antimicrobial-resistance potential (Taur et al., 2012; Peled et al., 2020; Yu et al., 2021; Messina et al., 2021).

A review focused only on dysbiosis is now too imprecise. The clinically important question is which microbial functions are lost, how those losses translate into host injury, and whether they are preventable or reversible. The Research Topic of dietary fiber, microbiota stability and host health provides an especially useful lens. Dietary fiber is the major luminal substrate supporting microbial fermentation and short-chain fatty acid production, and microbial responses to fiber are linked to mucus integrity, epithelial metabolism, immune signaling and systemic inflammatory tone (Trompette et al., 2014; Sonnenburg et al., 2016; Desai et al., 2016; Makki et al., 2018; Wastyk et al., 2021). In leukemia, fiber deprivation is rarely a simple dietary preference. It is often a consequence of nausea, mucositis, bowel rest, infection-control diets, parenteral nutrition and hospital food restrictions. These exposures occur exactly when barrier repair and immune recovery are most needed.

The present manuscript therefore treats the gut microbiota as a therapeutic ecology system. Resilience means more than taxonomic diversity. It includes resistance to domination, preservation of anaerobic metabolic networks, maintenance of colonization resistance, recovery after antibiotics, and sustained production of metabolites that support epithelial and immune homeostasis. This framing connects adult leukemia care to broader cancer microbiome principles while respecting the distinctive risks of neutropenia and transplantation (Secombe et al., 2021; Sepich-Poore et al., 2021; Oh et al., 2021; Guevara-Ramírez et al., 2023; Guevara-Ramírez et al., 2024). It also sets a boundary around intervention claims. Probiotics, prebiotics, fecal microbiota transplantation and metabolite strategies may be useful, but they require timing, patient selection and safety monitoring that differ from general-population nutrition advice.

## 2 Review scope and evidence base

We documented a PubMed evidence-mapping search performed on 13 May 2026 and covering publications from 13 May 2021 to 13 May 2026. The search combined leukemia/leukaemia/leukemic terms, AML, ALL, CLL and CML terms with gut microbiota, gut microbiome, intestinal microbiota, intestinal microbiome, gut flora, intestinal flora, dysbiosis, microbiota and microbiome. Of 276 initial PubMed records, 62 pediatric- or adolescent-focused records were excluded, leaving 214 adult or non-pediatric records for public full-text retrieval. Open-access full text was obtained for 164 unique records, represented by 167 downloaded files, and 137 unique full-text records were entered into the editable evidence matrix after local parsing and relevance mapping. The complete PubMed search string, public full-text retrieval route and PRISMA-style screening flow are provided in Supplementary file S1, in which the flow diagram is presented as Supplementary Figure S1. The complete editable record-level evidence matrix is provided separately as Supplementary Table S1.

**Figure 1:**
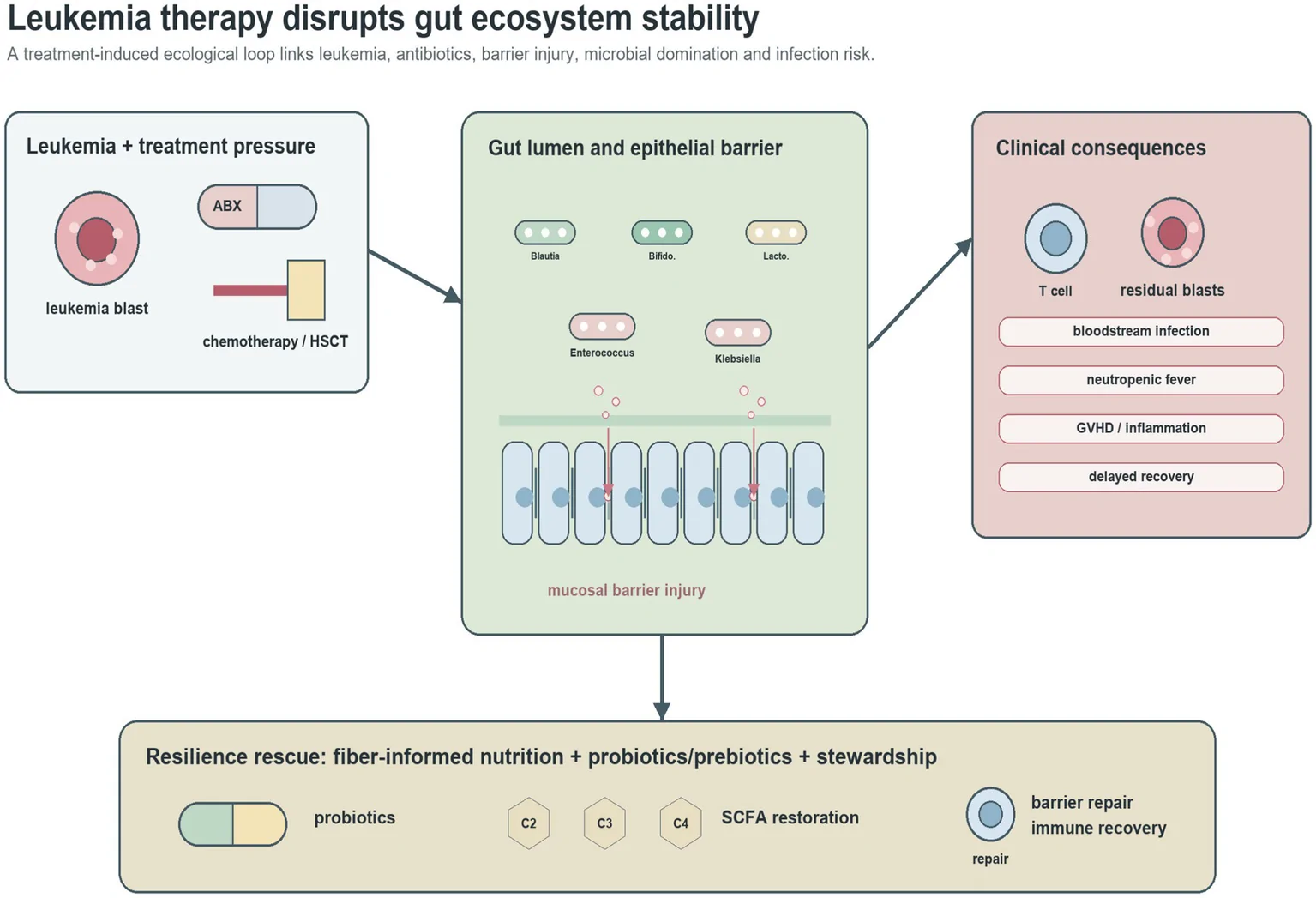
Treatment-driven collapse of gut ecosystem stability in leukemia. Leukemia burden, chemotherapy, antibiotics, neutropenia, and HCT-related injury reduce anaerobic diversity, favor pathobiont domination, and damage the epithelial barrier. The lower intervention axis summarizes a safety-bounded resilience strategy: preserve fermentable substrate, support probiotic or prebiotic restoration when appropriate, and integrate antimicrobial stewardship to promote SCFA recovery, barrier repair, and immune recovery.

**Figure 2:**
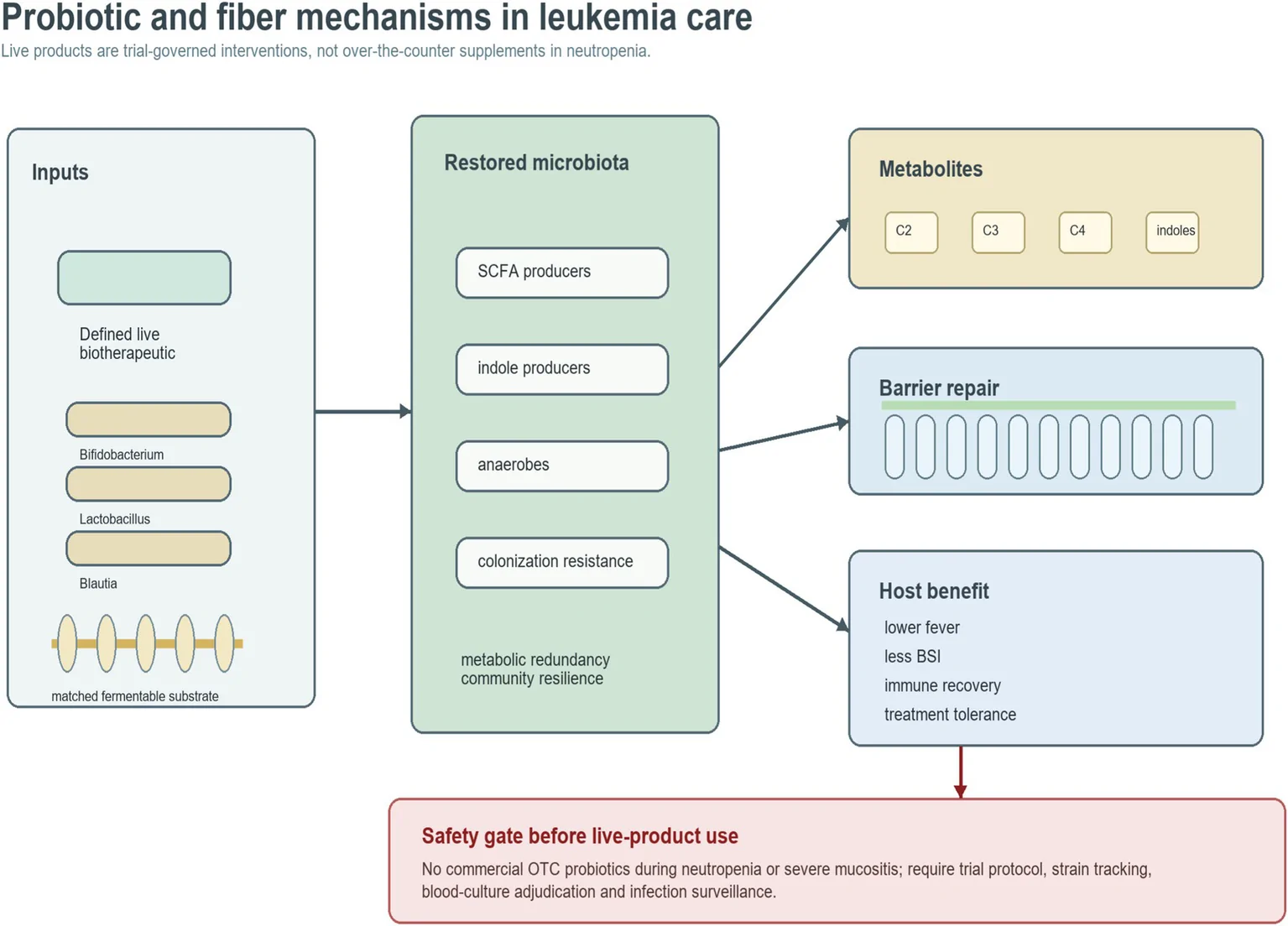
Fiber-supported probiotic and commensal mechanisms in leukemia care. *Bifidobacterium*, *Lactobacillus* and other commensal or live biotherapeutic candidates are placed within a substrate-dependent ecosystem, rather than as stand-alone supplements. The revised schematic links fermentable fiber to metabolic redundancy, colonization resistance, SCFA and indole signaling, epithelial repair, immune regulation, treatment tolerance and a safety gate requiring clinical-trial governance, strain tracking and bloodstream-infection surveillance.

**Figure 3:**
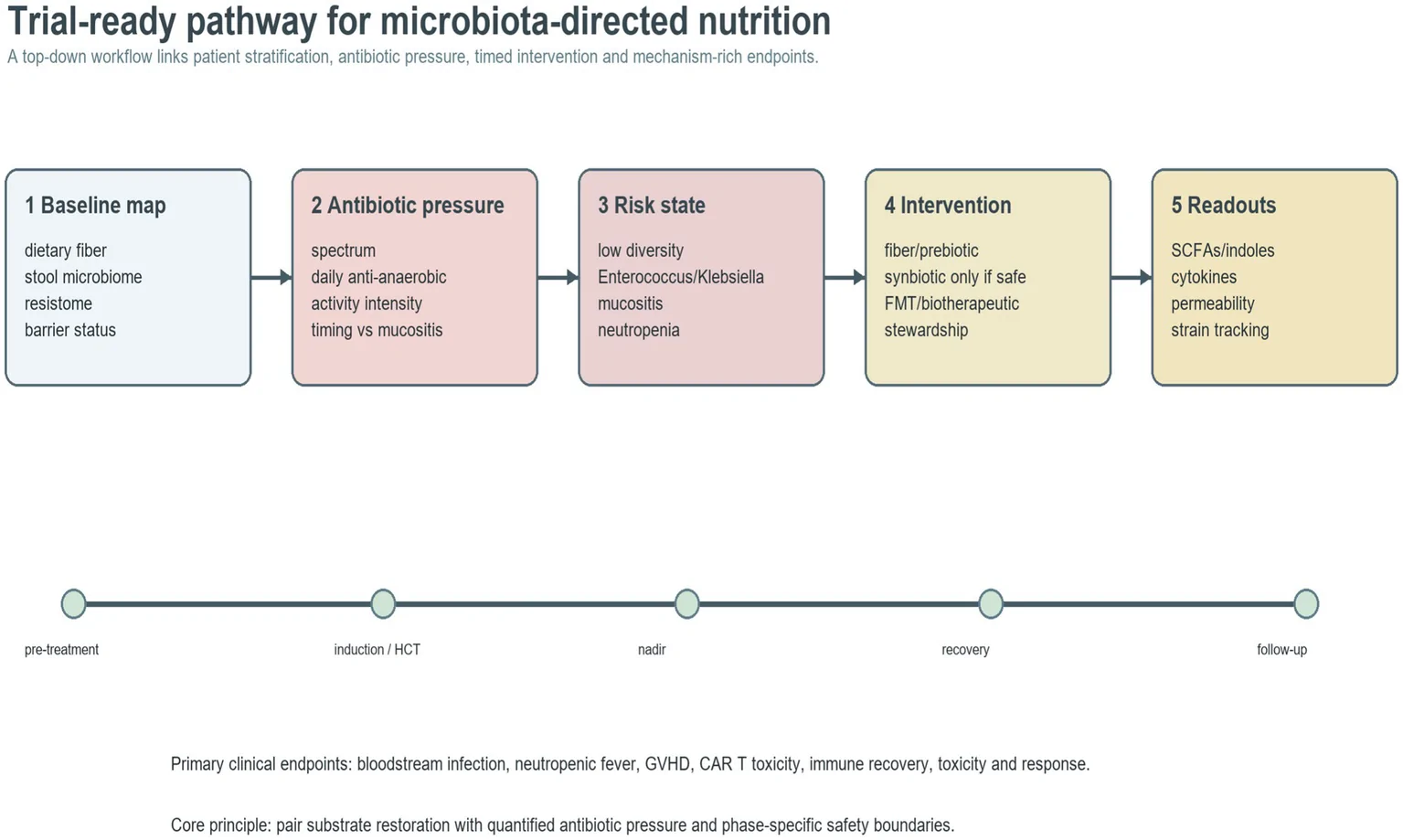
Trial-ready framework for microbiota-directed nutrition and therapeutic ecology. Baseline diet, microbiome state, antibiotic pressure, daily anti-anaerobic activity intensity, and barrier status define risk states. Intervention windows are aligned with treatment phase, and readouts connect microbiota restoration to metabolites, permeability, strain tracking, infection, immune recovery, toxicity, and response.

The evidence matrix was constructed to enhance transparency in evidence mapping rather than to serve as a quantitative data-extraction instrument. For each full-text article, we recorded the PMID, publication year, article type, leukemia-related context, full-text source, evidence category, principal mechanistic domains and relevance to the review framework. This structure enabled the provenance and inferential weight of each cited evidence stream to be assessed according to whether it derived from direct adult leukemia studies, adjacent human hematology cohorts, preclinical or mechanistic investigations, causal-inference analyses or broader microbiome literature (Figures 1–3).

To make this appraisal explicit, we added a qualitative study-assessment layer to the evidence map. Each study was judged by directness to adult leukemia, human versus preclinical design, prospective or retrospective ascertainment, sample size, microbiome method, exposure timing, clinical endpoint definition and control for major confounders. We did not apply a numerical risk-of-bias score because this Review integrates heterogeneous observational cohorts, trials, mechanistic studies and causal-inference analyses rather than pooling effect estimates. Instead, studies with high clinical directness and time-resolved exposure data were given greater inferential weight, while small cross-sectional, animal-only or non-leukemia studies were used only to support mechanism or hypothesis generation (Tables 1–3).

**Table 1:** Adult leukemia and transplantation settings in which microbiota stability is clinically relevant.

Clinical setting	Dominant ecological pressure	Key microbial or metabolic signal	Clinical implication
AML at diagnosis	Leukemia inflammation, anorexia, medication exposure and altered host metabolism before treatment.	Altered composition, SCFA and amino-acid pathway changes, reduced functional reserve.	Baseline diet and microbiome phenotyping should precede induction whenever feasible.
AML induction chemotherapy	Mucositis, neutropenia, empiric antibiotics, reduced intake and hospitalization.	Loss of protective metabolites, Enterococcus domination, SCFA-producer depletion and oral-gut coalescence.	Microbiota trajectories may help stratify fever, bloodstream infection, resistant colonization and recovery risk.
Neutropenic enterocolitis	Severe epithelial injury with microbial translocation risk.	Barrier disruption combined with loss of anaerobic colonization resistance.	Fiber and live products require phase-specific caution; recovery-phase substrate restoration may be testable.
Allogeneic HCT and GVHD	Conditioning injury, alloimmunity, antibiotics, diet restriction and immunosuppression.	Low diversity, domination, metabolite depletion, Blautia-associated protection and diet-sensitive recovery.	Nutrition, stewardship and microbiota restoration should be tested as integrated GVHD-modifying exposures.
CAR T-cell therapy	Immune effector activation, lymphodepletion, antibiotics and inflammation.	Microbiome features associated with response, cytokine release syndrome and toxicity.	Microbiota may influence immune efficacy, not only infection risk.
CLL and CML	Chronic immune dysregulation, age, comorbidity, targeted therapy and outpatient diet patterns.	Disease-associated diversity patterns, medication signatures and T-cell exhaustion mechanisms.	Useful models for long-term diet-microbiota-immune interactions.
Microbiota interventions	Attempted recovery after antibiotics, chemotherapy or transplant injury.	FMT engraftment, pooled fecal microbiotherapy, probiotic candidates, resistome changes.	Safety endpoints must include bacteremia, resistant organisms, drug exposure and unexpected immune toxicity.

**Table 2:** Mechanistic pathways linking dietary fiber-responsive microbiota to leukemia outcomes.

Pathway	Microbial function	Host mechanism	Leukemia-relevant outcome
Fermentable substrate flow	Fiber supports anaerobic cross-feeding and metabolic redundancy.	Maintains ecological resilience and recovery after perturbation.	Reduced probability of alternative stable states dominated by pathobionts.
SCFA production	Butyrate, propionate and acetate from fiber-fermenting taxa.	Colonocyte energy, tight junctions, mucus support and immune regulation.	Barrier repair, lower translocation risk and improved treatment tolerance.
Indole and bile-acid signaling	Tryptophan and bile-acid transformation by specialized microbes.	Epithelial regeneration, interferon signaling, mTOR/STING pathways and immune tone.	Potential modulation of GVHD, mucositis and antileukemia immune balance.
Colonization resistance	Diverse anaerobic community limits Enterococcus, Gram-negative and resistant-organism expansion.	Nutrient competition, pH modulation, niche exclusion and immune conditioning.	Lower bloodstream infection and resistant colonization risk.
Barrier-immune coupling	Microbial metabolites and structural signals shape mucosal and systemic immunity.	Myeloid recovery, T-cell function, inflammatory cytokines and epithelial repair.	Neutropenic fever, GVHD, CAR T toxicity and immune recovery endpoints.
Pharmacomicrobiomics	Microbial enzymes and community state alter drug metabolism or exposure.	Changed absorption, activation, inactivation, toxicity or pharmacodynamics.	Need for pharmacokinetic monitoring during microbiota restoration.
Dietary pattern and survivorship	Long-term substrate quality, fat exposure and lifestyle factors shape community structure.	Metabolic inflammation, immune aging and recovery after therapy.	Potential survivorship target in chronic leukemia and post-treatment recovery.

**Table 3:** Recommended endpoints for full-scale trials of fiber-informed microbiota interventions in adult leukemia.

Endpoint domain	Recommended measures	Why it is required
Dietary exposure	Daily fiber grams, fermentable fiber type, soluble/insoluble fraction, resistant starch or inulin-type substrate, diet quality, oral intake interruptions, enteral/parenteral nutrition.	Defines substrate exposure and avoids reducing the intervention to the nonspecific advice to eat more whole grains.
Antibiotic pressure	Spectrum, anaerobic activity, timing relative to chemotherapy/mucositis, duration, prophylaxis, escalation, de-escalation and daily anti-anaerobic activity intensity.	Controls the dominant ecological confounder and quantifies the exposure most likely to override fiber-mediated microbiota restoration.
Microbial ecology	Longitudinal diversity, domination, strain tracking, engraftment, anaerobe depletion and recovery time.	Measures resilience rather than one-time dysbiosis.
Resistome and pathogens	Resistance genes, VRE/CRE/Pseudomonas colonization, oral-gut coalescence and bloodstream isolates.	Links ecological change to infection and antimicrobial-resistance risk.
Live biotherapeutic safety	Blood-culture adjudication, strain-level matching of administered organisms, fungemia/bacteremia surveillance and serious-adverse-event review.	Prevents probiotic or live-biotherapeutic trials from being interpreted as low-risk supplementation in neutropenia.
Metabolomics	Fecal and plasma SCFAs, indoles, bile-acid derivatives, amino-acid products and microbial drug metabolites.	Tests functional mechanisms downstream of diet and microbial composition.
Barrier and inflammation	Mucositis grade, diarrhea, permeability markers, epithelial injury markers and cytokines.	Connects microbial function to host injury and immune activation.
Pharmacology	Drug exposure, toxicity, response and pharmacodynamic markers for gut-exposed agents.	Detects beneficial or harmful drug-microbe interactions.
Clinical outcomes	Neutropenic fever, bloodstream infection, enterocolitis, GVHD, CAR T toxicity, immune recovery, response, hospital days and survival.	Ensures ecological restoration translates into patient-centered benefit.

Accordingly, evidence was not treated as uniform in strength or clinical applicability. Direct adult leukemia clinical and translational studies were used as the primary basis for leukemia-specific clinical associations. HCT and mixed hematologic-malignancy cohorts were interpreted as adjacent human evidence, particularly for GVHD, immune reconstitution, infection risk, antibiotic pressure and safety-related endpoints. Preclinical and mechanistic studies were used to support biological plausibility, including pathways involving butyrate-dependent barrier integrity, the leukemia immune microenvironment, CD8 + T-cell exhaustion and microbial modulation of drug metabolism (Wang et al., 2022; ElMokh et al., 2022; Renga et al., 2024; Ronchi et al., 2026). Causal-inference and genetic studies were considered supportive triangulating evidence, whereas broader microbiome and dietary-fiber literature was incorporated only when it clarified mechanisms with direct relevance to leukemia care.

Contradictory or non-concordant findings were handled by separating the level of inference rather than averaging across designs. For example, consistent associations between low diversity, domination, antibiotic pressure and adverse outcomes were interpreted as clinically important signals when observed in adult leukemia or HCT cohorts, whereas differences in individual taxa across studies were not treated as definitive because they may reflect geography, diet, sequencing platform, antibiotic policy, disease phase and hospitalization. The public full-text restriction was used to ensure that population, methods, exposure timing, limitations and supplementary data could be manually verified during drafting; it was not intended to imply that inaccessible articles lack relevance.

The review also distinguishes adult leukemia from adjacent contexts. HCT, GVHD and CAR T-cell therapy were included because many adult leukemia patients receive these therapies and because the microbiota literature is richest in those settings. Chronic lymphocytic leukemia and chronic myeloid leukemia were included where studies examined disease-associated microbiota, medication effects or immune mechanisms. Pediatric-only studies were not used to support adult clinical claims. This boundary is important because the microbiota, diet, treatment intensity, antibiotic exposure and immune-developmental context differ substantially between children and adults.

This hierarchy was used to calibrate the strength of translational claims. Fiber, prebiotics, live biotherapeutics, FMT, immune recovery, infection reduction and pharmacomicrobiomics are described as clinical associations only when supported by adult leukemia or closely related human hematology evidence. When support is mainly preclinical, transplant-adjacent or indirect, the same topics are framed as mechanisms to test, safety boundaries to respect or endpoints for prospective trials.

Three questions organize the synthesis. First, which fiber-responsive microbial functions appear most relevant to adult leukemia outcomes? Second, which treatment exposures push the ecosystem toward low-resilience states? Third, which intervention windows are plausible enough for clinical testing without exceeding the safety boundaries of neutropenia, mucositis and transplantation? This structure is intended to give readers a field map, not a catalogue of isolated microbiome associations.

## 3 Leukemia as an ecological stress test

### 3.1 Disease-associated microbial remodeling before therapy

Microbiota instability may begin before the first dose of induction chemotherapy. Adult myeloid leukemia cohorts have reported altered gut microbial profiles at diagnosis, and the abnormalities often coexist with anorexia, muscle weakness, inflammatory features and metabolomic shifts (Yu et al., 2021; Pötgens et al., 2024; An et al., 2025). These findings matter because they challenge the assumption that the pretreatment microbiota is a healthy baseline. Leukemia itself can reshape host metabolism, immune tone, oral intake and mucosal physiology, while prediagnostic antibiotics, proton-pump inhibitors, comorbidities and dietary changes may further alter the community. A stool sample collected at admission therefore represents a disease-conditioned ecological state, not a neutral control.

Several adult AML studies converge on metabolic disturbance rather than a single pathognomonic taxon. Microbiome and metabolome analyses have described changes in SCFAs, carnosine-histidine metabolism, amino-acid pathways and microbial community structure (Xu et al., 2023; Wu et al., 2023; Rashidi et al., 2022b). These alterations plausibly interact with appetite, muscle function, barrier integrity and inflammation. Importantly, such studies are not yet sufficient to infer causality or prescribe a universal microbial signature. The more durable message is that adult leukemia is accompanied by a shift in microbial function, and that baseline functional capacity may influence how well patients tolerate treatment-related perturbation.

### 3.2 Chemotherapy, mucositis and neutropenia

Induction chemotherapy converts microbial vulnerability into clinical risk. Cytotoxic injury damages rapidly dividing epithelium, reduces mucosal barrier integrity and creates a niche in which bacteria and microbial products can cross the mucosa. Neutropenia then limits containment of translocation events. At the same time, nausea, anorexia and mucositis reduce enteral intake, while empiric broad-spectrum antibiotics remove anaerobic competitors and select for organisms that tolerate hospitalization and antimicrobial pressure. Studies of acute leukemia patients show that altered microbiota-host metabolic crosstalk can precede neutropenic fever, that protective microbial metabolites decline after fever, and that SCFA-producing microbes can help distinguish infectious from non-infectious fever states (Rashidi et al., 2021; Rashidi et al., 2022a; Franklin et al., 2026).

Enterococcus domination illustrates this ecological cascade. In acute leukemia chemotherapy populations, intestinal domination by Enterococcus has been associated with higher mortality, while oral-stool coalescence and bloodstream infection studies suggest that chemotherapy and antibiotics blur the boundary between oral, intestinal and systemic microbial compartments (Messina et al., 2021; Franklin et al., 2022; McMahon et al., 2023). These observations should not be reduced to a simple pathogen story. Enterococcus expansion is a marker of lost colonization resistance, altered nutrient competition, antibiotic selection and epithelial disruption. It tells clinicians that the ecosystem has lost checks and balances that normally prevent a single organism from occupying a dangerous fraction of the community.

### 3.3 Transplantation and GVHD

Allogeneic HCT magnifies the same processes. Conditioning injures epithelium, antibiotics restructure the microbiota, donor immune cells encounter microbial products, and GVHD can further amplify barrier damage. Foundational studies linked intestinal domination and low diversity to bacteremia and mortality, and recent HCT reviews and cohorts extend those findings to microbial metabolites, immune recovery, diet and prospective intervention designs (Taur et al., 2012; Peled et al., 2020; Wolfe and Markey, 2022; van Lier et al., 2023; Yue et al., 2024). The HCT literature is therefore central to adult leukemia microbiome research even when the study population includes multiple hematologic malignancies.

The fiber-microbiota connection is particularly visible in transplantation because the clinical pathway often includes dietary restriction, reduced oral intake, parenteral nutrition and antibacterial exposure. A recent study comparing sterile and normal dietary exposure after HCT showed that sterile diet can collapse gut microbiome recovery while normal diet supports restoration (Hong et al., 2024). This does not prove that every patient should receive high-fiber food during severe mucositis, but it strongly argues that nutrition policy is not ecologically neutral. Diet, antibiotics and immune suppression should be modeled as interacting exposures rather than separate supportive-care details.

## 4 Fiber-dependent microbial resilience

### 4.1 From fermentable substrate to alternative stable states

Fermentable fiber sustains microbial resilience by providing substrate to anaerobes that generate SCFAs and support cross-feeding networks. In healthy or stable states, functional redundancy allows different organisms to maintain similar metabolic outputs despite small perturbations. Leukemia care threatens this redundancy on multiple fronts: reduced dietary fiber lowers substrate supply, antibiotics remove sensitive anaerobes, mucositis changes host nutrient leakage, and hospitalization favors organisms adapted to oxygen, inflammation and antibiotic pressure. The ecosystem may then enter an alternative stable state in which low diversity, low fermentation and pathobiont domination reinforce one another (Makki et al., 2018; Sonnenburg et al., 2016; Desai et al., 2016).

This concept explains why microbiota recovery can lag behind count recovery. Longitudinal AML studies have shown lasting shifts in the gut microbiota after therapy, and recovered acute leukemia cohorts can continue to display altered intestinal microbiota composition (Rashidi et al., 2022c; Vázquez et al., 2024). In practical terms, discharge from hospital does not necessarily mark ecological recovery. A patient may have normalized neutrophil counts yet remain metabolically depleted, dominated by facultative taxa or colonized with resistant organisms. Interventions designed only for the nadir may miss an important recovery window when substrate, live biotherapeutics or microbiota restoration could help rebuild function.

### 4.2 SCFAs and epithelial repair

Butyrate provides the strongest mechanistic bridge between dietary fiber and leukemia outcomes. It is a major energy source for colonocytes, supports tight-junction function, influences mucus biology and modulates immune responses. In AML, a mechanistic study showed that gut microbiota can regulate leukemia through alteration of intestinal barrier function mediated by butyrate (Wang et al., 2022). Newly diagnosed AML studies further report SCFA changes, and neutropenic fever studies implicate depletion of SCFA-producing microbes and protective metabolites (An et al., 2025; Franklin et al., 2026; Rashidi et al., 2022a). The evidence does not mean that butyrate is a stand-alone drug for leukemia, but it does establish barrier metabolism as a plausible mediator between microbial ecology and clinical outcome.

We further clarified that clinical SCFA measurements are not equivalent to microbial production. Fecal concentrations reflect the net balance of substrate intake, microbial synthesis, epithelial uptake, luminal dilution, transit time, stool water content and mucosal inflammation. Because butyrate is largely consumed locally by colonocytes, a fecal butyrate value may underestimate epithelial exposure in one patient and reflect failed absorption or barrier injury in another. For this reason, SCFAs are presented as functional biomarkers that require paired diet, metagenomic, metabolomic, barrier and antibiotic data rather than as stand-alone surrogates of microbial health.

SCFA biology also provides a rational endpoint framework. Trials should measure fecal and plasma acetate, propionate and butyrate, but they should not interpret them in isolation. SCFA concentrations reflect intake, microbial capacity, transit, epithelial absorption and inflammation. A low stool butyrate signal may indicate depleted butyrate producers, altered consumption by injured epithelium or both. Therefore, SCFA endpoints should be paired with diet records, microbial gene or strain profiles, barrier markers, mucositis grades and antibiotic exposure. Such integrated measurement is more likely to identify actionable resilience states than single-time-point diversity indices.

### 4.3 Indoles, bile acids and amino-acid metabolites

The fiber-microbiota axis should not be narrowed to SCFAs alone. Tryptophan-derived indoles, bile-acid metabolites and amino-acid products are increasingly implicated in epithelial repair, immune regulation, GVHD biology and leukemia metabolism. In transplantation, metabolite-focused work links microbial products to intestinal regeneration and alloimmune injury. Desaminotyrosine, for example, has been reported to protect against GVHD through mTORC1 and STING-dependent intestinal regeneration in mechanistic studies (Göttert et al., 2025). AML studies have also identified carnosine-histidine and amino-acid metabolic pathways, while bile-acid-related work suggests that microbial-metabolic pathways can intersect with leukemia cell biology and macrophage polarization (Wu et al., 2023; Liu et al., 2022b).

These metabolite pathways are clinically important because they may respond differently to diet and antibiotics. Some indole-producing organisms are highly sensitive to broad anaerobic coverage; bile-acid transformation depends on specialized microbial enzymes; and amino-acid metabolites may reflect both host catabolism and microbial function. In adult leukemia trials, broad metabolomics can therefore reveal whether a fiber-informed intervention restores a coherent metabolic network or merely increases one analyte. It can also identify unexpected risk. For instance, microbial succinic semialdehyde has been reported to promote proliferation of adult T-cell leukemia/lymphoma cells in experimental work, reminding the field that microbial metabolites are context-dependent signals rather than universally protective molecules (Chiba et al., 2024).

## 5 Disease-specific evidence across the leukemia spectrum

### 5.1 Acute myeloid leukemia

AML is the most mature adult leukemia model for microbiota research. Cohorts at diagnosis, longitudinal treatment datasets, mechanistic studies and recent reviews collectively show that AML is associated with altered microbial composition, metabolite disruption, barrier dysfunction and treatment-related ecological collapse (Yu et al., 2021; Rashidi et al., 2022c; Rashidi et al., 2022b; Pötgens et al., 2024; Ji et al., 2025; Alvaro et al., 2026). The field has moved from broad diversity comparisons toward mechanistic questions: which taxa and functions predict neutropenia duration, fever, infection, chemotherapy resistance, mucositis or immune recovery? Recent work linking gut microbiota to chemotherapy-induced neutropenia duration and antibiotic-resistant events illustrates this transition from descriptive profiling to risk stratification (Huang et al., 2025; Franklin et al., 2025; McMahon et al., 2025).

Chemotherapy resistance and drug response are emerging AML priorities. A 2025 review specifically emphasized microbiome dysbiosis and chemotherapy resistance in AML, while pharmacomicrobiomic reviews describe multiple routes by which microbial communities may shape drug efficacy and toxicity (Tian et al., 2025; Nowicka et al., 2025). The evidence is not yet ready for routine clinical decision-making, but it has already changed the conceptual model. The gut microbiota can influence treatment not only by modifying infection risk, but also by changing barrier integrity, systemic inflammation, immune tone, metabolite availability and direct microbial drug metabolism.

The acute promyelocytic leukemia and AML preclinical literature illustrates both promise and caution. *Bifidobacterium pseudolongum* administered with arsenic trioxide attenuated acute promyelocytic leukemia in mice by restoring immune microenvironment and intestinal homeostasis (Guo et al., 2026). Curcumin sensitized AML response to cytarabine in association with intestinal microbiota regulation, and NAD-lowering therapy was hampered by the gut microbiota in leukemia models (Liu et al., 2022a; ElMokh et al., 2022). These studies should be interpreted as mechanistic probes rather than direct adult clinical recommendations. They do, however, show that microbial ecology can interact with antileukemic therapy in ways that are biologically plausible and therapeutically relevant.

### 5.2 Acute leukemia complications: fever, enterocolitis and infection

Neutropenic fever is one of the most clinically actionable microbiota endpoints in acute leukemia. Studies have shown altered microbiota-host metabolic crosstalk before fever, loss of protective metabolites after fever and microbial signatures that distinguish infectious from non-infectious fever states (Rashidi et al., 2021; Rashidi et al., 2022a; Franklin et al., 2026). These data suggest that fever is not only a host event detected at the thermometer; it may be preceded by measurable ecological and metabolic changes. If confirmed prospectively, such signals could help identify patients who need intensified surveillance, earlier de-escalation, targeted nutrition or microbiota restoration.

Neutropenic enterocolitis makes the barrier dimension explicit. Recent work combining gut microbiota and intestinal barrier analyses in neutropenic enterocolitis supports the view that mucosal injury, microbial disruption and systemic infection risk are inseparable (Kapandji et al., 2025). A fiber-informed framework is useful here but must be clinically cautious. During severe enterocolitis, aggressive fiber administration may be unsafe. During prevention or recovery, however, preserving enteral substrate and anaerobic metabolism could be important for barrier repair. This argues for timed intervention windows rather than a single nutrition rule for all phases of leukemia care.

### 5.3 HCT, GVHD and immune reconstitution

HCT studies provide the clearest clinical evidence linking microbiota state to hard outcomes. Low diversity, domination and microbial metabolite loss have been associated with mortality, bacteremia, GVHD and immune recovery in multiple cohorts and reviews (Peled et al., 2020; Zargari Marandi et al., 2022; Wolfe and Markey, 2022; van Lier et al., 2023; Yue et al., 2024). Protective taxa such as Blautia have been linked to lower neutropenic fever risk in allogeneic transplant recipients, whereas Enterococcus expansion and diet-sensitive microbial collapse represent loss of colonization resistance (Rashidi et al., 2022d; Stein-Thoeringer et al., 2019; Hong et al., 2024). The mechanistic interpretation is consistent: microbial communities influence the epithelial and immune terrain on which alloreactivity unfolds.

At the same time, HCT and GVHD evidence cannot be directly transposed to leukemia induction or consolidation therapy. Conditioning intensity, donor-derived immunity, calcineurin inhibitors, systemic corticosteroids, GVHD-associated diarrhea, antifungal exposure, nutritional route and prolonged antibacterial therapy create an ecological setting that differs from non-transplant AML treatment. We therefore use HCT data to define adjacent human hematology mechanisms and safety endpoints, while leukemia-specific clinical claims are anchored to adult leukemia cohorts whenever available.

FMT and microbiota restoration studies have begun to test causality in HCT-related contexts. A randomized double-blind phase II trial compared FMT with placebo in allogeneic HCT and AML, while multi-omics analysis of an FMT trial identified new aspects of acute GVHD pathogenesis (Rashidi et al., 2023; Rashidi et al., 2024b). The FMT-allo and MAST protocols show that controlled microbiota transplantation is moving into prospective testing with safety oversight and mechanistic endpoints (Dougé et al., 2023; Mullish et al., 2024). These trials are pivotal because they ask whether rebuilding the ecosystem changes clinical biology, not simply whether dysbiosis predicts outcomes.

Dietary fat and dietary pattern studies also matter in transplantation. Animal-fat exposure has been reported to disrupt gut microbiota and aggravate sclerodermatous chronic GVHD after allogeneic stem cell transfer (Millick et al., 2026). Together with sterile-diet data, this supports the broader principle that diet can shape post-transplant immune injury. The translational message is not to replace immunosuppression with diet. It is that diet composition, enteral access, microbial substrate flow and immune injury should be integrated in GVHD prevention and recovery studies.

### 5.4 CAR T-cell therapy and lymphoid leukemia

CAR T-cell therapy extends microbiota relevance beyond conventional chemotherapy and HCT, but the evidence remains mostly associative and should be integrated cautiously into the fiber-barrier-immunity axis. Human studies link gut microbiome features with anti-CD19 CAR T-cell response, cytokine release syndrome and toxicity, while experimental work suggests that microbiota modulation can enhance antigen cross-presentation and antitumor CAR T-cell activity (Smith et al., 2022; Hu et al., 2022; Uribe-Herranz et al., 2023). Mechanistically, microbial metabolites such as SCFAs and tryptophan derivatives may influence T-cell differentiation, histone acetylation, cytokine tone and epithelial antigen handling; however, direct proof in adult leukemia CAR T cohorts is still limited.

CLL and CML provide complementary chronic models. In CLL, microbiome diversity, B-cell receptor inhibitor exposure, poor-outcome signatures and machine-learning discrimination of disease or medication status suggest that the microbiota may interact with age, comorbidity, myeloid-cell activation and T-cell exhaustion rather than act only through acute infection risk (Faitová et al., 2022; Zuccaro et al., 2023; Faitova et al., 2024; Paziewska et al., 2024; Fait Kadlec et al., 2025). In CML, preclinical work linking low-abundance intestinal bacteria to MyD88/TRIF-dependent CD8 + T-cell exhaustion supports a plausible immune-metabolic route (Ronchi et al., 2026). These observations strengthen the conceptual coherence of the fiber-barrier-immunity axis, but they remain hypothesis-generating until prospective disease-specific studies measure diet, antibiotics, metabolites and immune phenotypes together.

CLL provides a different model: chronic immune dysregulation, long-term medication exposure and outpatient management. Adult CLL studies have described gut microbiome alterations, associations with B-cell receptor inhibitor treatment, microbial diversity links to CLL development, poor-outcome signatures and machine-learning identification of CLL and medication use based on microbiome data (Faitová et al., 2022; Zuccaro et al., 2023; Faitova et al., 2024; Paziewska et al., 2024; Fait Kadlec et al., 2025). The evidence remains smaller than in AML, and confounding by age, diet, comorbidity and medication is substantial. Even so, CLL may be valuable for studying durable interactions among diet, microbial ecology, immune senescence and targeted therapy.

CML and related chronic leukemias add further mechanistic breadth. Low-abundance intestinal bacteria have been reported to drive MyD88/Trif-dependent CD8 T-cell exhaustion in chronic myeloid leukemia, suggesting that small community components can have disproportionate immunologic effects (Ronchi et al., 2026). Causal-inference studies across hematologic malignancies and leukemia also report associations among gut microbiota, immune cells, inflammatory proteins and serum metabolites (Chen et al., 2023; Chen et al., 2024; Luo et al., 2024; Zhuang et al., 2024; Zhu et al., 2025). Mendelian randomization cannot replace clinical trials, but it can prioritize microbial-host axes for mechanistic validation.

## 6 Clinical consequences of microbiota instability

### 6.1 Colonization resistance, bloodstream infection and the resistome

The most immediate clinical consequence of microbiota instability is loss of colonization resistance. A diverse anaerobic ecosystem suppresses pathobiont expansion through nutrient competition, metabolic by-products, pH changes, bacteriocins, spatial exclusion and immune conditioning. When antibiotics and low substrate availability remove these pressures, Enterococcus, Klebsiella, Pseudomonas and other hospital-adapted organisms may expand. Oral and gastrointestinal microbiome studies in leukemia patients show that organisms associated with bloodstream infection can be traced across mucosal compartments, supporting a translocation and ecological-source model (McMahon et al., 2023; Franklin et al., 2022; Messina et al., 2021).

Antibiotic resistance adds a second layer. AML studies have begun to characterize antibiotic administration factors associated with microbiome disruption and subsequent antibiotic-resistant infection or colonization events, and machine-learning models are being developed to predict such outcomes (Franklin et al., 2025; McMahon et al., 2025). Broader hematology studies link gut microbiome features and resistome elements to colonization and infection with antibiotic-resistance threats (Batool et al., 2025). These data make antibiotic stewardship part of microbiota therapeutics. A fiber or probiotic intervention cannot be evaluated properly unless the investigators know whether anaerobic niches are being simultaneously removed by antimicrobial exposure.

FMT and microbiota restoration also intersect with antimicrobial resistance. Randomized data outside leukemia show that FMT can have long- and short-term effects on antibiotic resistance genes, and hematology-specific real-world data suggest that FMT may be used to address resistant Pseudomonas colonization in selected patients (Rashidi et al., 2024a; Nowicka and Gil, 2026). These findings are promising but safety-sensitive. Donor screening, resistance-gene surveillance, strain tracking and infection adjudication should be mandatory when donor-derived or live products are used in neutropenic or post-transplant populations.

### 6.2 Immune recovery and systemic inflammation

Microbiota effects on leukemia outcomes are not restricted to bacteria entering the bloodstream. Microbial metabolites influence epithelial sensing, myeloid cell education, T-cell polarization, interferon pathways and hematopoietic tone. The HCT immune-recovery literature explicitly links the intestinal microbiome to immune reconstitution, while diet-targeted human studies outside leukemia show that microbiota-directed diets can modulate immune status (Wolfe and Markey, 2022; Wastyk et al., 2021). In leukemia, the key translational question is whether preserving fermentative metabolism can reduce maladaptive inflammation while allowing antileukemia immunity to recover.

The answer is likely phase-specific. During induction, the priority may be barrier preservation, fever risk reduction and avoidance of resistant domination. During immune recovery, microbial signals may shape myeloid regeneration, lymphocyte function and vaccine responsiveness. During transplantation, the same signals may influence the balance between GVHD and graft-versus-leukemia effects. This is why one-size-fits-all microbiota restoration is unlikely to succeed. The desired immune state differs across induction, consolidation, HCT, CAR T-cell therapy and chronic targeted treatment.

### 6.3 Pharmacomicrobiomics and treatment response

Pharmacomicrobiomics has become a central reason to study the leukemia microbiota. Microbial communities can metabolize drugs directly, alter epithelial absorption, change enterohepatic circulation, influence inflammation-mediated pharmacodynamics and modify treatment toxicity. Recent AML and blood-cancer pharmacomicrobiomics reviews describe this emerging field in detail (Nowicka et al., 2025; Hsu et al., 2026). Specific studies show gut bacterial O-demethylation can modulate systemic exposure to oral etoposide, CPX-351 can exploit the gut microbiota to promote mucosal barrier function and immune homeostasis, and microbial ecology can interfere with NAD-lowering therapy (Tripathi et al., 2026; Renga et al., 2024; ElMokh et al., 2022).

The clinical implication is double-edged. Restoring anaerobic function may improve barrier integrity and immune recovery, but it may also alter drug exposure, toxicity or efficacy. This is especially relevant for oral agents, maintenance therapy, older AML regimens, targeted drugs and prolonged outpatient therapy. Trials of fiber, probiotics, FMT, phage therapy or engineered microbial products should therefore include pharmacokinetic or pharmacodynamic endpoints when interventions overlap with gut-exposed therapies. Otherwise, a biologically active microbiota intervention could be misclassified as safe or ineffective because drug-microbe interactions were not measured.

We also separated demonstrated drug-microbiome interactions from plausible but unproven effects of microbiota restoration. Evidence that bacteria can alter oral etoposide exposure, influence CPX-351-associated barrier biology or affect NAD-targeted therapy is stronger than the hypothesis that fiber, FMT or a live biotherapeutic will improve leukemia pharmacokinetics. The trial implication is therefore not to assume benefit, but to measure drug exposure, toxicity, response and microbial function whenever a microbiota-directed intervention overlaps with gut-exposed antileukemic therapy.

Microbiota-inspired therapeutics further complicate the boundary between supportive care and antileukemic therapy. A microbiota-inspired synthetic compound targeting NPM1 has been identified through cancer-stem-cell phenotype-guided discovery, and engineered microbiome approaches are being proposed for blood cancer immunotherapy (Algar et al., 2024; Cheng et al., 2025). These directions are early, but they show that the field is moving from passive biomarkers toward designed microbial and metabolite interventions. For clinical hematologists, this demands a high standard of mechanistic evidence, safety monitoring and patient selection.

## 7 Microbiota-directed interventions

### 7.1 Nutrition and dietary fiber

Dietary fiber is the intervention most aligned with the target Research Topic, but it should be treated as a clinical exposure with dose, chemical structure, timing and tolerance rather than as a vague healthy-diet label. In this Review, dietary fiber refers to non-digestible carbohydrate delivered by foods, medical nutrition or formulas; fermentable substrate or prebiotic refers to a selectively utilized compound such as inulin-type fructans, fructo-oligosaccharides, galacto-oligosaccharides or resistant starch; and synbiotic refers to an intentional pairing of a live organism with a complementary substrate (Makki et al., 2018). In adult leukemia, the same substrate may be useful before treatment, poorly tolerated during severe mucositis, and testable again during recovery.

This distinction also prevents the oversimplified message that leukemia patients should merely increase whole-grain intake. A natural food fiber matrix, purified inulin-type fructan, fructo-oligosaccharide, galacto-oligosaccharide, resistant starch and medically formulated enteral substrate differ in viscosity, fermentability, gas production, osmotic load and microbial niche requirements. Their feasibility is also phase-dependent: a substrate that is plausible before induction may be poorly tolerated during severe mucositis or neutropenic enterocolitis and may become testable again during epithelial recovery.

Causal-inference work has begun to connect dietary factors, gut microbiota, host metabolites and AML risk, and lifestyle reviews in hematological cancer survivors highlight diet, activity and microbiota as survivorship questions (Qin et al., 2025; Samami et al., 2025). These studies do not yet define a leukemia-specific fiber prescription. They do, however, justify prospective trials that record actual intake, use mechanistically chosen substrates and measure microbial and host outputs. A reasonable first objective is not to prove that fiber cures leukemia; it is to test whether preserving fermentable substrate reduces loss of SCFA-producing communities, barrier injury, domination and infection risk.

Clinical nutrition teams should be central to such studies. Leukemia patients often experience dysgeusia, nausea, diarrhea, mucositis, neutropenic dietary restrictions and anxiety about food safety. A trial that ignores tolerability and food delivery will fail regardless of microbiology. Practical interventions may include individualized fiber-preserving menus, medically supervised enteral formulas with defined fermentable substrates, gradual reintroduction during recovery and monitoring for bloating, diarrhea, ileus or enterocolitis. The research design should capture missed meals and oral-intake interruptions as carefully as it captures sequencing reads.

Hospital dietary restrictions deserve explicit ecological scrutiny. Historically, low-microbial or sterile diets were designed to prevent infection during profound immunosuppression, but their clinical benefit is incompletely supported and they may reduce the substrate flow needed for anaerobic recovery. The HCT study showing microbiome collapse with sterile diet and recovery with normal diet supports the principle that safe normal or fiber-preserving nutrition can be an ecological intervention, while still requiring phase-specific adaptation for mucositis, enterocolitis and food-safety risk (Hong et al., 2024).

### 7.2 Probiotics, prebiotics and live biotherapeutics

Probiotics and live biotherapeutics are attractive because they offer a visible way to restore function, but they are also the area where hematologists must be most disciplined. Bifidobacterium and Lactobacillus appear in mechanistic and preclinical leukemia studies, and Blautia has been associated with protection against neutropenic fever after allogeneic transplantation (Guo et al., 2026; Rashidi et al., 2022d). These signals support controlled research, but they do not justify over-the-counter probiotic use during neutropenia, severe mucositis, active GVHD or uncontrolled bacteremia. A reported *Lactobacillus rhamnosus* bloodstream infection in a hematology patient, supported by metagenomic strain comparison, illustrates that live organisms can become clinically dangerous in the exact population most likely to be considered for microbiota restoration (Mannavola et al., 2025).

The next generation of live biotherapeutics should therefore be function-defined and trial-governed. Rather than selecting organisms because they are commercially familiar, trials should ask whether a product restores butyrate production, indole metabolism, bile-acid transformation, colonization resistance, immune modulation or antibiotic-resistance suppression. Protocols should include strain tracking, blood-culture adjudication, serious-adverse-event review and predefined stopping rules. Prebiotics may be safer in some settings, but only if the relevant microbial niche remains present. Synbiotic designs, pairing substrate with a defined organism or consortium, should be tested only after the patient’s mucosal barrier, neutrophil status and antimicrobial exposure make live-product risk acceptable.

### 7.3 FMT, pooled fecal microbiotherapy, phage and engineered approaches

FMT and pooled fecal microbiotherapy have moved from rescue anecdotes toward controlled hematology trials. FMT has been studied after allogeneic HCT and AML, protocols are testing FMT to prevent post-transplant complications, and oral pooled fecal microbiotherapy after intensive chemotherapy has shown feasibility in a phase 1b trial (Rashidi et al., 2023; Dougé et al., 2023; Mullish et al., 2024; Malard et al., 2025). Case-based reports of washed microbiota transplantation or salvage FMT in complex post-transplant or lymphocytic leukemia settings further demonstrate clinical interest, although they cannot establish efficacy (Zhang et al., 2024; Finotto et al., 2025).

The safety framework for donor-derived products must be stricter in leukemia than in most gastroenterology populations. Donor screening should include multidrug-resistant organisms, emerging pathogens and resistance genes; recipients should be stratified by neutropenia, mucositis, GVHD, central line status and immunosuppression; and endpoints should include bacteremia, fungemia, resistant colonization, unexpected inflammatory syndromes and drug exposure. Phage therapy and microbiome-directed anti-resistance strategies are also being discussed in hematologic malignancies, but early evidence should be regarded as hypothesis-generating (Zhang et al., 2025). The appeal is precision: rather than replacing the entire ecosystem, phage or engineered approaches could reduce a dangerous organism while preserving anaerobic resilience.

### 7.4 Antibiotic stewardship as microbiota therapy

Antibiotic stewardship deserves equal status with probiotics and FMT in leukemia microbiome trials. The antibiotic is often the dominant ecological intervention, and its effect cannot be captured by a simple exposed/unexposed variable. Spectrum, anaerobic activity, timing relative to chemotherapy and mucositis, days of therapy, prophylaxis, escalation during fever, de-escalation and cumulative exposure all shape the microbiota (Franklin et al., 2022; Franklin et al., 2025). Future studies should therefore calculate a daily anti-anaerobic activity intensity or comparable antibiotic-pressure metric and use it as a primary stratification variable, not merely as a *post hoc* covariate. Broad ecological harm must still be balanced against the immediate need to treat infection in a high-risk patient; the scientific goal is not antibiotic avoidance but measurable stewardship.

Stewardship also interacts with diet. A fermentable substrate can only support anaerobic recovery if organisms capable of using it survive or can re-engraft. Conversely, a restricted diet may worsen the ecological consequences of antibiotics by removing the substrate needed for recovery. Trials should therefore prespecify antibiotic strata or use adaptive designs that account for anti-anaerobic exposure. Clinical protocols could test whether early de-escalation, narrower empiric regimens when safe, or post-antibiotic microbiota restoration improves recovery of SCFA-producing communities without increasing infection-related mortality.

Mechanistically, substrate availability and antibiotic pressure should be modeled as an interaction rather than as two independent exposures. Anti-anaerobic antibiotics can remove the organisms and cross-feeding partners required to convert fiber into butyrate, propionate, indoles and secondary bile-acid products. In a severely depleted ecosystem, additional fermentable substrate may pass through unused, increase symptoms or preferentially feed facultative pathobionts. Therefore, fiber-mediated restoration should be interpreted only in relation to residual anaerobic capacity, re-engraftment potential and daily anti-anaerobic activity intensity.

## 8 A full-review trial framework for the fiber-microbiota-leukemia axis

A mature trial framework should begin before treatment. Baseline assessment should include leukemia subtype, treatment plan, comorbidities, body composition, appetite, oral health, medication exposure, diet history, recent antibiotics, stool microbiome, resistome and metabolome. Sampling should continue through induction or conditioning, nadir, fever episodes, antibiotic changes, mucositis peak, count recovery, discharge and outpatient follow-up. The objective is to map ecological trajectories, not to capture a single stool snapshot. This is especially important because leukemia therapy is a sequence of perturbations rather than one exposure.

Mechanistic endpoints should include strain-level or metagenomic profiling, functional pathway analysis, fecal and plasma SCFAs, indoles, bile acids and amino-acid metabolites, epithelial injury markers, permeability markers, inflammatory cytokines, neutrophil and lymphocyte recovery, immune-cell phenotyping and microbiologically adjudicated infections. Trial stratification should include baseline diet, microbiome state, mucositis or barrier status, and daily anti-anaerobic activity intensity. Clinical endpoints should include neutropenic fever, bloodstream infection, resistant-organism colonization, Clostridioides difficile infection, enterocolitis, GVHD, CAR T-cell toxicity, response, hospital days and survival.

Trial designs should be phase-specific. Before induction or HCT, an optimization study could test whether fiber-preserving nutrition improves baseline SCFA-producing capacity. During treatment, a prevention study could test whether controlled enteral substrate and antibiotic stewardship reduce domination and fever. During recovery, a restoration study could test prebiotics, synbiotics, defined live biotherapeutics or donor-derived microbiotherapy in patients with persistent low diversity or resistant colonization. During chronic therapy, a pragmatic outpatient study could examine diet, medication and microbiota interactions in CLL or CML. The same intervention should not be assumed to fit all phases.

Patient selection is crucial. A patient with preserved anaerobic diversity, tolerable oral intake and low pathobiont burden may be a good candidate for fiber-enhancing nutrition. A patient with severe mucositis, ileus, enterocolitis or uncontrolled bacteremia is not. A patient with persistent domination after antibiotics may need staged restoration, first addressing safety and antimicrobial pressure, then introducing substrate or live products. A patient receiving oral etoposide or another gut-exposed drug may require pharmacokinetic monitoring if microbiota restoration is introduced. Precision should be defined by ecological state, not by enthusiasm for a product class.

## 9 Clinical implementation and safety

For clinicians, the immediate lesson is to document microbiota-relevant exposures. Daily diet quality, fiber intake, route of nutrition, mucositis grade, diarrhea, proton-pump inhibitor use, laxatives, antifungals, antibacterial spectrum and fever episodes should be recorded in a way that can be analyzed. Without this, microbiome data remain difficult to interpret. A patient with low diversity after induction may have leukemia-related dysbiosis, antibiotic-driven collapse, starvation of fermentative taxa, mucositis-driven nutrient leakage or all of these. High-quality clinical annotation is a prerequisite for high-quality microbiome science.

Safety should be framed around predictable leukemia risks. Commercial probiotics should be discouraged outside validated clinical trials in patients with neutropenia, severe mucositis, active GVHD, uncontrolled bloodstream infection or central-line sepsis concern. Function-defined live biotherapeutics require bacteremia and fungemia surveillance, strain-level attribution when bloodstream infection occurs, donor or product resistome screening, and independent adjudication of serious infections. Fiber interventions require monitoring for intolerance, ileus or enterocolitis, and pharmacomicrobiomic interventions require toxicity and exposure monitoring. The field should avoid two extremes: dismissing microbiota interventions as too risky for hematology, or assuming that natural products are automatically safe.

Multidisciplinary implementation is essential. Hematologists understand disease phase and infection risk; infectious disease specialists and pharmacists understand antimicrobial pressure; dietitians understand substrate delivery and tolerability; microbiologists and computational biologists understand strain tracking and function; transplant and cellular therapy teams understand immune complications. The fiber-microbiota barrier axis sits at the intersection of these disciplines. Its translation will fail if treated as a supplement decision made outside the leukemia care pathway.

## 10 Knowledge gaps

Several gaps now define the field. First, adult leukemia lacks adequately powered randomized trials that directly manipulate dietary fiber while measuring microbial stability, metabolites, barrier function and clinical outcomes. Second, resilience is inconsistently defined. Diversity, domination, strain persistence, metabolic output and recovery time are related but not interchangeable. Third, antibiotic exposure is often measured too crudely, obscuring diet and microbiota effects. Fourth, leukemia subtype and treatment phase are sometimes pooled in ways that hide biologically important differences. Fifth, most studies remain underpowered for hard outcomes such as bloodstream infection, GVHD, treatment response and survival.

Methodological gaps are equally important. Stool sampling does not fully capture mucosal communities, metabolomics varies by collection and processing, and diet records are often missing or imprecise. Many studies use 16S sequencing when strain-level transmission, resistome and functional-pathway questions require metagenomics. Confounding by medications, disease severity, hospitalization and diet is difficult to eliminate. Causal-inference studies can prioritize hypotheses, but they require mechanistic validation and prospective clinical testing (Chen et al., 2023; Chen et al., 2024; Yang et al., 2024; Zhu et al., 2025). These limitations should motivate better trials, not therapeutic nihilism.

Reverse causation is a central challenge in this field. Chemotherapy, fever, mucositis, anorexia, hospital diet, parenteral nutrition, proton-pump inhibitors, antifungals, opioids, laxatives, transfusion intensity, disease severity and length of hospitalization can all reshape the microbiota while also predicting infection, toxicity and survival. Future studies should therefore collect time-stamped exposure data and avoid interpreting a single dysbiotic sample as evidence that dysbiosis preceded the clinical event.

Two leukemia-specific causal gaps deserve priority. The first is the bidirectional relation between mucositis and microbiota: dysbiosis may worsen mucosal injury by depleting SCFAs and colonization resistance, but chemotherapy-induced epithelial exudate, bleeding, mucus loss and reduced intake may also reshape the nutritional landscape that selects the microbiota. The second is reverse causation in GVHD: microbial disruption may contribute to GVHD risk, while GVHD therapy, especially high-dose corticosteroids and intensified antibiotics, can itself profoundly remodel the microbial community.

Finally, the field needs clinically meaningful definitions of success. Restoring alpha diversity is not enough if infection, mucositis, GVHD or toxicity do not improve. Conversely, a targeted intervention that reduces domination, restores butyrate production or lowers resistant colonization may be valuable even if global diversity changes modestly. Future studies should align endpoints with mechanism: fiber trials should measure fermentative output and barrier repair; stewardship trials should measure anaerobe preservation and resistant events; FMT trials should measure engraftment, safety and clinical complications; pharmacomicrobiomic trials should measure drug exposure and toxicity.

Prospective designs should therefore collect time-resolved samples before, during and after mucositis, antibiotic escalation, steroid initiation and cellular or transplant therapy. Without this temporal structure, causal-inference models risk mistaking treatment consequences for microbial causes. The most informative trials will pair ecological readouts with treatment-phase anchors rather than relying on a single stool sample or a single diversity metric.

## 11 Conclusion

Adult leukemia care exposes the gut microbiota to one of the strongest perturbation environments in medicine. The recent full-text literature supports a coherent model in which leukemia biology, chemotherapy, HCT, mucosal injury, neutropenia, antibiotics and nutritional disruption converge to reduce microbiota stability, deplete protective metabolites, impair barrier integrity, weaken colonization resistance and alter immune and pharmacologic outcomes. Dietary fiber belongs near the top of this model because it supplies the substrate for microbial functions that maintain epithelial and immune resilience. Yet in leukemia, fiber must be handled with the precision of a clinical intervention. The right substrate, patient, phase and safety boundary matter.

The next stage of the field should move from observing dysbiosis to managing therapeutic ecology. This requires trials that integrate nutrition, antimicrobial stewardship, microbiome function, metabolomics, barrier biology, immune recovery and patient-centered outcomes. It also requires humility. Microbiota restoration can plausibly improve leukemia care, but it can also transfer organisms, change drug exposure or behave differently across disease phases. The practical endpoint is therefore not a probiotic label or a diversity score. It is a monitored program that preserves fermentative anaerobic function when possible, identifies high-risk ecological states early, restores microbial functions safely during recovery and tests whether these changes improve infection, immune and treatment outcomes.

## References

[ref1] Algar S. Vázquez-Villa H. Aguilar-Garrido P. Navarro-Aguadero M. Á. Velasco-Estévez M. Sánchez-Merino A. . (2024). Cancer-stem-cell phenotype-guided discovery of a microbiota-inspired synthetic compound targeting NPM1 for leukemia. JACS Au 4, 1786–1800. doi: 10.1021/jacsau.3c00682, 38818079 PMC11134387

[ref2] Alvaro M. E. Caserta S. Martino E. A. Skafi M. Bruzzese A. Amodio N. . (2026). Gut microbiota and acute myeloid leukemia: state of the art, clinical signals, and translational opportunities. Antibiotics 15:417. doi: 10.3390/antibiotics15040417, 42041380 PMC13113732

[ref3] An S. Gong X. Zhao L. Jian J. Guo Y. Yang X. . (2025). Significant changes in gut microbiota and SCFAs among patients with newly diagnosed acute myeloid leukemia. Front. Microbiol. 16:1559033. doi: 10.3389/fmicb.2025.1559033, 40236478 PMC11997447

[ref4] Batool M. McMahon S. Franklin S. Ramont C. Sahasrabhojane P. Chang C. C. . (2025). Gut microbiome features and resistome elements associated with colonization and infection with antibiotic-resistance threats. Gut Microbes Rep 2:2570502. doi: 10.1080/29933935.2025.2570502, 41909910 PMC12940128

[ref5] Chen P. Guo J. Wang W. Feng A. Qin L. Hu Y. . (2024). Refining the relationship between gut microbiota and common hematologic malignancies: insights from a bidirectional Mendelian randomization study. Front. Cell. Infect. Microbiol. 14:1412035. doi: 10.3389/fcimb.2024.1412035, 38975324 PMC11224959

[ref6] Chen G. Kuang Z. Li F. Li J. (2023). The causal relationship between gut microbiota and leukemia: a two-sample Mendelian randomization study. Front. Microbiol. 14:1293333. doi: 10.3389/fmicb.2023.1293333, 38075916 PMC10703164

[ref7] Cheng F. Soleimani Samarkhazan H. Khazaei Y. (2025). CRISPR-engineered microbiome: living therapeutics revolutionize blood cancer immunotherapy. NPJ Biofilms Microbiomes 12:17. doi: 10.1038/s41522-025-00882-9, 41387457 PMC12816696

[ref8] Chiba N. Suzuki S. Enriquez-Vera D. Utsunomiya A. Kubuki Y. Hidaka T. . (2024). Succinic semialdehyde derived from the gut microbiota can promote the proliferation of adult T-cell leukemia/lymphoma cells. Heliyon. 10:e38507. doi: 10.1016/j.heliyon.2024.e38507, 39640675 PMC11619966

[ref9] Desai M. S. Seekatz A. M. Koropatkin N. M. Kamada N. Hickey C. A. Wolter M. . (2016). A dietary fiber-deprived gut microbiota degrades the colonic mucus barrier and enhances pathogen susceptibility. Cell 167, 1339–1353.e21. doi: 10.1016/j.cell.2016.10.043, 27863247 PMC5131798

[ref10] Dougé A. Ravinet A. Corriger A. Cabrespine A. Wasiak M. Pereira B. . (2023). Faecal microbiota transplantation to prevent complications after allogeneic stem cell transplantation for haematological malignancies: a study protocol for a randomised controlled phase-II trial (the FMT-Allo study). BMJ Open 13:e068480. doi: 10.1136/bmjopen-2022-068480, 37130682 PMC10163541

[ref11] ElMokh O. Matsumoto S. Biniecka P. Bellotti A. Schaeuble K. Piacente F. . (2022). Gut microbiota severely hampers the efficacy of NAD-lowering therapy in leukemia. Cell Death Dis. 13:320. doi: 10.1038/s41419-022-04763-3, 35396381 PMC8993809

[ref12] Fait Kadlec T. Ilett E. E. da Cunha-Bang C. Sengeløv H. Brieghel C. Gulay A. . (2025). Explainable machine learning to identify chronic lymphocytic leukemia and medication use based on gut microbiome data. Microbiol. Spectrum 13:e0094425. doi: 10.1128/spectrum.00944-25, 41196057 PMC12671114

[ref13] Faitova T. Coelho M. Da Cunha-Bang C. Ozturk S. Kartal E. Bork P. . (2024). The diversity of the microbiome impacts chronic lymphocytic leukemia development in mice and humans. Haematologica 109, 3237–3250. doi: 10.3324/haematol.2023.284693, 38721725 PMC11443378

[ref14] Faitová T. Svanberg R. Da Cunha-Bang C. Ilett E. E. Jørgensen M. Noguera-Julian M. . (2022). The gut microbiome in patients with chronic lymphocytic leukemia. Haematologica 107, 2238–2243. doi: 10.3324/haematol.2021.280455, 35548869 PMC9425300

[ref15] Finotto T. Chevenet C. Fayard A. (2025). Fecal microbiota transplantation as a salvage therapy for concomitant resistant digestive graft versus host disease and cryptosporidiosis in a patient post hematopoietic stem cell transplant: about a case. Mediter J Hematol Infect Dis 17:e2025035. doi: 10.4084/MJHID.2025.035, 40375906 PMC12081037

[ref16] Franklin S. Aitken S. L. Shi Y. Sahasrabhojane P. V. Robinson S. Peterson C. B. . (2022). Oral and stool microbiome coalescence and its association with antibiotic exposure in acute leukemia patients. Front. Cell. Infect. Microbiol. 12:848580. doi: 10.3389/fcimb.2022.848580, 35433514 PMC9010033

[ref17] Franklin S. Ramont C. Batool M. McMahon S. Sahasrabhojane P. Blazier J. C. . (2025). Characterization of antibiotic administration factors associated with microbiome disruption and subsequent antibiotic-resistant infection and colonization events in acute myeloid leukemia patients receiving chemotherapy. Antibiotics 14:770. doi: 10.3390/antibiotics14080770, 40867965 PMC12382674

[ref18] Franklin S. Sahasrabhojane P. Hayase T. Hayase E. Chang C. C. Senapati J. . (2026). Short-chain fatty acid-producing microbes differentiate non-infectious and infectious neutropenic fever in leukemia. mSystems 11:e01343-25. doi: 10.1128/msystems.01343-25, 41914747 PMC13098215

[ref19] Göttert S. Thiele Orberg E. Fan K. Heinrich P. Matthe D. M. Khalid O. . (2025). The microbial metabolite desaminotyrosine protects against graft-versus-host disease via mTORC1 and STING-dependent intestinal regeneration. Nat. Commun. 16:9282. doi: 10.1038/s41467-025-65180-6, 41115965 PMC12537870

[ref20] Guevara-Ramírez P. Cadena-Ullauri S. Paz-Cruz E. Ruiz-Pozo V. A. Tamayo-Trujillo R. Cabrera-Andrade A. . (2024). Gut microbiota disruption in hematologic cancer therapy: molecular insights and implications for treatment efficacy. Int. J. Mol. Sci. 25:10255. doi: 10.3390/ijms251910255, 39408584 PMC11476909

[ref21] Guevara-Ramírez P. Cadena-Ullauri S. Paz-Cruz E. Tamayo-Trujillo R. Ruiz-Pozo V. A. Zambrano A. K. (2023). Role of the gut microbiota in hematologic cancer. Front. Microbiol. 14:1185787. doi: 10.3389/fmicb.2023.1185787, 37692399 PMC10485363

[ref22] Guo Z. Gao Z. Zhao Y. Ni X. Zhang W. Li L. . (2026). Administering *Bifidobacterium pseudolongum* with arsenic trioxide attenuates acute *Promyelocytic leukemia* in mice by restoring immune microenvironment and intestinal homeostasis. Fron Biosci 31:48584. doi: 10.31083/FBL48584, 41761979

[ref23] Hong W. Wu Y. Sun Z. Yang S. Cheng Q. Liu H. . (2024). Sterile diet causes gut microbiome collapse of cancer patients post hematopoietic cell transplantation, but normal diet recovers them. Adv. Sci. 11:2403991. doi: 10.1002/advs.202403991, 38973355 PMC11425903

[ref24] Hsu C. Y. Almajidi Y. Q. Abohassan M. Gafarov R. Basunduwah T. S. Hjazi A. . (2026). Pharmacomicrobiomics in blood cancers: how the gut microbiome and its metabolites shape drug efficacy and toxicity. Crit. Rev. Oncol. Hematol. 221:105229. doi: 10.1016/j.critrevonc.2026.105229, 41740635

[ref25] Hu Y. Li J. Ni F. Yang Z. Gui X. Bao Z. . (2022). CAR-T cell therapy-related cytokine release syndrome and therapeutic response is modulated by the gut microbiome in hematologic malignancies. Nat. Commun. 13:5313. doi: 10.1038/s41467-022-32960-3, 36085303 PMC9461447

[ref26] Huang Y. Liao L. Jiang Y. Tao S. Tang D. (2025). Role of gut microbiota in predicting chemotherapy-induced neutropenia duration in leukemia patients. Front. Microbiol. 16:1507336. doi: 10.3389/fmicb.2025.1507336, 40177485 PMC11961871

[ref27] Ji M. Ji M. Zhong Y. Shao L. (2025). Gut microbiota in acute myeloid leukemia: from biomarkers to interventions. Meta 15:568. doi: 10.3390/metabo15090568, 41002952 PMC12471731

[ref28] Kapandji N. Salmona M. Lemoine A. Ulmann G. Calderaro J. Roche B. . (2025). Unravelling neutropenic enterocolitis: insights from gut microbiota, and intestinal barrier analyses. Exp. Hematol. Oncol. 14:74. doi: 10.1186/s40164-025-00661-4, 40380332 PMC12084932

[ref29] Liu J. Luo W. Chen Q. Chen X. Zhou G. Sun H. (2022a). Curcumin sensitizes response to cytarabine in acute myeloid leukemia by regulating intestinal microbiota. Cancer Chemother. Pharmacol. 89, 243–253. doi: 10.1007/s00280-021-04385-0, 35066694 PMC8807457

[ref30] Liu J. Wei Y. Jia W. Can C. Wang R. Yang X. . (2022b). Chenodeoxycholic acid suppresses AML progression through promoting lipid peroxidation via ROS/p38 MAPK/DGAT1 pathway and inhibiting M2 macrophage polarization. Redox Biol. 56:102452. doi: 10.1016/j.redox.2022.102452, 36084349 PMC9465103

[ref31] Luo C. Zhang W. Zhu J. Qiu T. Fang Q. (2024). Interleukin-2 mediated associations between gut microbiota and acute myeloid leukemia: a population-based mediation Mendelian randomization study. Heliyon. 10:e33194. doi: 10.1016/j.heliyon.2024.e33194, 39022041 PMC11252755

[ref32] Makki K. Deehan E. C. Walter J. Backhed F. (2018). The impact of dietary fiber on gut microbiota in host health and disease. Cell Host Microbe 23, 705–715. doi: 10.1016/j.chom.2018.05.012, 29902436

[ref33] Malard F. Thepot S. Cluzeau T. Carré M. Lebon D. Bories P. . (2025). Gut microbiota restoration with oral pooled fecal microbiotherapy after intensive chemotherapy: the phase 1b CIMON trial. Blood Adv. 9, 3739–3749. doi: 10.1182/bloodadvances.2024015571, 40197991 PMC12305571

[ref34] Mannavola C. M. De Maio F. Marra J. Fiori B. Santarelli G. Posteraro B. . (2025). Bloodstream infection by *Lactobacillus rhamnosus* in a haematology patient: why metagenomics can make the difference. Gut Pathog. 17:47. doi: 10.1186/s13099-025-00722-3 40544256 PMC12182651

[ref35] McMahon S. Franklin S. Galloway-Peña J. (2025). Utilization of machine learning to predict antibiotic resistant event outcomes in acute myeloid leukemia patients undergoing induction chemotherapy. Front. Cell. Infect. Microbiol. 15:1629422. doi: 10.3389/fcimb.2025.1629422, 40918253 PMC12408608

[ref36] McMahon S. Sahasrabhojane P. Kim J. Franklin S. Chang C. C. Jenq R. R. . (2023). Contribution of the Oral and gastrointestinal microbiomes to bloodstream infections in leukemia patients. Microbiol. Spectrum 11:e0041523. doi: 10.1128/spectrum.00415-23, 37022173 PMC10269818

[ref37] Messina J. A. Tan C. Y. Ren Y. Hill L. Bush A. Lew M. . (2021). Enterococcus intestinal domination is associated with increased mortality in the acute leukemia chemotherapy population. Clin. Infect. Dis. 78, 414–422. doi: 10.1093/cid/ciab1043, 34928341 PMC10874263

[ref38] Millick D. D. Ghosh S. Wu G. Krzyzanowska A. K. Osorio L. Zhao L. . (2026). Dietary animal fat disrupts gut microbiota and aggravates Scl-cGVHD after allogeneic hematopoietic stem cell transfer. Blood Adv. 10, 1204–1216. doi: 10.1182/bloodadvances.2024014831, 41191542 PMC12917525

[ref39] Mullish B. H. Innes A. J. Roberts L. A. Anim-Burton S. Webber L. Johnson N. A. . (2024). Intestinal microbiota transplant prior to allogeneic stem cell transplant (MAST) trial: study protocol for a multicentre, double-blinded, placebo-controlled, phase IIa trial. BMJ Open 14:e093120. doi: 10.1136/bmjopen-2024-093120, 39773995 PMC11884074

[ref40] Nowicka A. Gil L. (2026). Fecal microbiota transplantation for carbapenem-resistant *Pseudomonas* spp. colonization in hematology patients: long-term real-world data. Int. J. Infect. Dis. 165:108447. doi: 10.1016/j.ijid.2026.108447 41628815

[ref41] Nowicka A. Tomczak H. Szałek E. Karbownik A. Gil L. (2025). Microbial crosstalk with therapy: pharmacomicrobiomics in AML-one step closer to personalized medicine. Biomedicine 13:1761. doi: 10.3390/biomedicines13071761, 40722831 PMC12292961

[ref42] Oh B. Boyle F. Pavlakis N. Clarke S. Guminski A. Eade T. . (2021). Emerging evidence of the gut microbiome in chemotherapy: a clinical review. Front. Oncol. 11:706331. doi: 10.3389/fonc.2021.706331, 34604043 PMC8481611

[ref43] Paziewska M. Szelest M. Kiełbus M. Masternak M. Zaleska J. Wawrzyniak E. . (2024). Increased abundance of firmicutes and depletion of bacteroidota predicts poor outcome in chronic lymphocytic leukemia. Oncol. Lett. 28:552. doi: 10.3892/ol.2024.14685, 39328278 PMC11425030

[ref44] Peled J. U. Gomes A. L. C. Devlin S. M. Littmann E. R. Taur Y. Sung A. D. . (2020). Microbiota as predictor of mortality in allogeneic hematopoietic-cell transplantation. N. Engl. J. Med. 382, 822–834. doi: 10.1056/NEJMoa1900623, 32101664 PMC7534690

[ref45] Pötgens S. A. Havelange V. Lecop S. Li F. Neyrinck A. M. Bindels F. . (2024). Gut microbiome alterations at acute myeloid leukemia diagnosis are associated with muscle weakness and anorexia. Haematologica 109, 3194–3208. doi: 10.3324/haematol.2023.284138, 38546675 PMC11443375

[ref46] Qin J. Zhang L. Zhang G. Liao W. Yu L. (2025). Genetically predicted gut microbiota and host metabolites mediate the causal link between dietary factors and acute myeloid leukemia. Food Sci. Nutr. 13:e70456. doi: 10.1002/fsn3.70456, 40552330 PMC12183468

[ref47] Rashidi A. Ebadi M. Rehman T. U. Elhusseini H. Halaweish H. Holtan S. G. . (2022a). Loss of microbiota-derived protective metabolites after neutropenic fever. Sci. Rep. 12:6244. doi: 10.1038/s41598-022-10282-0, 35428797 PMC9012881

[ref48] Rashidi A. Ebadi M. Rehman T. U. Elhusseini H. Halaweish H. Kaiser T. . (2022b). Compilation of longitudinal gut microbiome, serum metabolome, and clinical data in acute myeloid leukemia. Sci. Data 9:468. doi: 10.1038/s41597-022-01600-2, 35918343 PMC9346123

[ref49] Rashidi A. Ebadi M. Rehman T. U. Elhusseini H. Halaweish H. F. Kaiser T. . (2022c). Lasting shift in the gut microbiota in patients with acute myeloid leukemia. Blood Adv. 6, 3451–3457. doi: 10.1182/bloodadvances.2021006783, 35192686 PMC9198907

[ref50] Rashidi A. Ebadi M. Rehman T. U. Elhusseini H. Kazadi D. Halaweish H. . (2023). Randomized double-blind phase II trial of fecal microbiota transplantation versus placebo in allogeneic hematopoietic cell transplantation and AML. J. Clin. Oncol. 41, 5306–5319. doi: 10.1200/JCO.22.02366, 37235836 PMC10691796

[ref51] Rashidi A. Ebadi M. Rehman T. U. Elhusseini H. Kazadi D. Halaweish H. . (2024a). Long- and short-term effects of fecal microbiota transplantation on antibiotic resistance genes: results from a randomized placebo-controlled trial. Gut Microbes 16:2327442. doi: 10.1080/19490976.2024.2327442, 38478462 PMC10939144

[ref52] Rashidi A. Ebadi M. Rehman T. U. Elhusseini H. Kazadi D. Halaweish H. . (2024b). Multi-omics analysis of a fecal microbiota transplantation trial identifies novel aspects of acute GVHD pathogenesis. Cancer Res Commun 4, 1454–1466. doi: 10.1158/2767-9764.CRC-24-0138, 38767452 PMC11164016

[ref53] Rashidi A. Ebadi M. Rehman T. U. Elhusseini H. Nalluri H. Kaiser T. . (2021). Altered microbiota-host metabolic cross talk preceding neutropenic fever in patients with acute leukemia. Blood Adv. 5, 3937–3950. doi: 10.1182/bloodadvances.2021004973, 34478486 PMC8945620

[ref54] Rashidi A. Peled J. U. Ebadi M. Rehman T. U. Elhusseini H. Marcello L. T. . (2022d). Protective effect of intestinal Blautia against neutropenic fever in allogeneic transplant recipients. Clin. Infect. Dis. 75, 1912–1920. doi: 10.1093/cid/ciac299, 35435976 PMC10200328

[ref55] Renga G. Nunzi E. Stincardini C. Pariano M. Puccetti M. Pieraccini G. . (2024). CPX-351 exploits the gut microbiota to promote mucosal barrier function, colonization resistance, and immune homeostasis. Blood 143, 1628–1645. doi: 10.1182/blood.2023021380, 38227935

[ref56] Ronchi F. Hinterbrandner M. Rubino V. Römmele M. Chiorazzo T. Mooser C. . (2026). Intestinal low-abundant bacteria drive MyD88/Trif-dependent CD8+ T cell exhaustion in chronic myeloid leukemia. Cell Rep. 45:116753. doi: 10.1016/j.celrep.2025.116753, 41442277

[ref57] Samami E. Starkweather A. Kelly D. L. Lyon D. (2025). The impact of health-promoting lifestyle behaviors on gut microbiota in survivors of hematological cancer: a scoping review. Cancer Rep. 8:70224. doi: 10.1002/cnr2.70224, 40364600 PMC12075932

[ref58] Secombe K. R. Al-Qadami G. H. Subramaniam C. B. Bowen J. M. Scott J. Van Sebille Y. Z. A. (2021). Guidelines for reporting microbiome studies in cancer: a systematic review. Crit. Rev. Oncol. Hematol. 160:103281. doi: 10.1016/j.critrevonc.2021.103281 33667660

[ref59] Sepich-Poore G. D. Zitvogel L. Straussman R. Hasty J. Wargo J. A. Knight R. (2021). The microbiome and human cancer. Science 371:eabc4552. doi: 10.1126/science.abc4552, 33766858 PMC8767999

[ref60] Smith M. Dai A. Ghilardi G. Amelsberg K. V. Devlin S. M. Pajarillo R. . (2022). Gut microbiome correlates of response and toxicity following anti-CD19 CAR T cell therapy. Nat. Med. 28, 713–723. doi: 10.1038/s41591-022-01702-9, 35288695 PMC9434490

[ref61] Sonnenburg E. D. Smits S. A. Tikhonov M. Higginbottom S. K. Wingreen N. S. Sonnenburg J. L. (2016). Diet-induced extinctions in the gut microbiota compound over generations. Nature 529, 212–215. doi: 10.1038/nature16504, 26762459 PMC4850918

[ref62] Stein-Thoeringer C. K. Nichols K. B. Lazrak A. Docampo M. D. Slingerland A. E. Slingerland J. B. . (2019). Lactose drives Enterococcus expansion to promote graft-versus-host disease. Science 366, 1143–1149. doi: 10.1126/science.aax3760, 31780560 PMC7003985

[ref63] Taur Y. Xavier J. B. Lipuma L. Ubeda C. Goldberg J. Gobourne A. . (2012). Intestinal domination and the risk of bacteremia in patients undergoing allogeneic hematopoietic stem cell transplantation. Clin. Infect. Dis. 55, 905–914. doi: 10.1093/cid/cis580, 22718773 PMC3657523

[ref64] Tian M. Soleimani Samarkhazan H. Alemohammad S. S. Manesh M. F. Tavakoli F. Shams A. . (2025). Microbiome dysbiosis and chemotherapy resistance in acute myeloid leukemia (AML). NPJ Biofilms Microbiomes 12:50. doi: 10.1038/s41522-025-00891-8, 41423676 PMC12909810

[ref65] Tripathi A. Kyawt T. E. Shin J. Won K. J. Armstrong A. T. Ilktach G. . (2026). Gut bacterial O-demethylation modulates systemic exposure to oral etoposide. Gut Microbes 18:2628358. doi: 10.1080/19490976.2026.2628358, 41688891 PMC12915777

[ref66] Trompette A. Gollwitzer E. S. Yadava K. Sichelstiel A. K. Sprenger N. Ngom-Bru C. . (2014). Gut microbiota metabolism of dietary fiber influences allergic airway disease and hematopoiesis. Nat. Med. 20, 159–166. doi: 10.1038/nm.3444, 24390308

[ref67] Uribe-Herranz M. Beghi S. Ruella M. Parvathaneni K. Salaris S. Kostopoulos N. . (2023). Modulation of the gut microbiota engages antigen cross-presentation to enhance antitumor effects of CAR T cell immunotherapy. Mol Therapy 31, 686–700. doi: 10.1016/j.ymthe.2023.01.012, 36641624 PMC10014349

[ref68] van Lier Y. F. Vos J. Blom B. Hazenberg M. D. (2023). Allogeneic hematopoietic cell transplantation, the microbiome, and graft-versus-host disease. Gut Microbes 15:2178805. doi: 10.1080/19490976.2023.2178805, 36794370 PMC9980553

[ref69] Vázquez X. Lumbreras-Iglesias P. Rodicio M. R. Fernández J. Bernal T. Moreno A. F. . (2024). Study of the intestinal microbiota composition and the effect of treatment with intensive chemotherapy in patients recovered from acute leukemia. Sci. Rep. 14:5585. doi: 10.1038/s41598-024-56054-w, 38454103 PMC10920697

[ref70] Wang R. Yang X. Liu J. Zhong F. Zhang C. Chen Y. . (2022). Gut microbiota regulates acute myeloid leukaemia via alteration of intestinal barrier function mediated by butyrate. Nat. Commun. 13:2522. doi: 10.1038/s41467-022-30240-8, 35534496 PMC9085760

[ref71] Wastyk H. C. Fragiadakis G. K. Perelman D. Dahan D. Merrill B. D. Yu F. B. . (2021). Gut-microbiota-targeted diets modulate human immune status. Cell 184, 4137.e14–4153.e14. doi: 10.1016/j.cell.2021.06.019, 34256014 PMC9020749

[ref72] Wolfe A. E. Markey K. A. (2022). The contribution of the intestinal microbiome to immune recovery after HCT. Front. Immunol. 13:988121. doi: 10.3389/fimmu.2022.988121, 36059482 PMC9434312

[ref73] Wu B. Xu Y. Tang M. Jiang Y. Zhang T. Huang L. . (2023). A metabolome and microbiome analysis of acute myeloid leukemia: insights into the carnosine-histidine metabolic pathway. Toxics 12:14. doi: 10.3390/toxics12010014, 38250970 PMC10821349

[ref74] Xu J. Kang Y. Zhong Y. Ye W. Sheng T. Wang Q. . (2023). Alteration of gut microbiome and correlated amino acid metabolism are associated with acute myelocytic leukemia carcinogenesis. Cancer Med. 12, 16431–16443. doi: 10.1002/cam4.6283, 37409640 PMC10469656

[ref75] Yang Q. Wang Z. Liu M. Gan L. (2024). Causal relationship between gut microbiota and leukemia: future perspectives. Oncol Therapy 12, 663–683. doi: 10.1007/s40487-024-00300-8, 39217582 PMC11573970

[ref76] Yu D. Yu X. Ye A. Xu C. Li X. Geng W. . (2021). Profiling of gut microbial dysbiosis in adults with myeloid leukemia. FEBS open bio. 11, 2050–2059. doi: 10.1002/2211-5463.13193, 33993646 PMC8406483

[ref77] Yue X. Zhou H. Wang S. Chen X. Xiao H. (2024). Gut microbiota, microbiota-derived metabolites, and graft-versus-host disease. Cancer Med. 13:e6799. doi: 10.1002/cam4.6799, 38239049 PMC10905340

[ref78] Zargari Marandi R. Jørgensen M. Ilett E. E. Nørgaard J. C. Noguera-Julian M. Paredes R. . (2022). Pre-transplant prediction of acute graft-versus-host disease using the gut microbiome. Cells 11:4089. doi: 10.3390/cells11244089, 36552852 PMC9776596

[ref79] Zhang X. Li Y. Li B. Wu J. Zhang J. Ding X. (2024). Washed microbiota transplantation in an elderly patient with lymphocytic leukemia and *Clostridioides difficile* infection: a case report illustrating a triumph over complexity. Heliyon. 10:e32450. doi: 10.1016/j.heliyon.2024.e32450, 39040423 PMC11260954

[ref80] Zhang J. Liu J. Bayani A. (2025). Phage therapy and the microbiome in hematologic malignancies: opportunities, mechanisms, and early evidence. J. Cancer Res. Clin. Oncol. 152:8. doi: 10.1007/s00432-025-06393-6, 41384994 PMC12701166

[ref81] Zhu L. Ruan X. Zou Y. Ye S. Xie L. (2025). The causal relationship between circulating inflammatory proteins, gut microbiotas, immune cells and leukemia: a bidirectional Mendelian randomization study. Discov. Oncol. 16:1157. doi: 10.1007/s12672-025-02863-y, 40537729 PMC12179026

[ref82] Zhuang X. Yin Q. Yang R. Man X. Wang R. Geng H. . (2024). Causal pathways in lymphoid leukemia: the gut microbiota, immune cells, and serum metabolites. Front. Immunol. 15:1437869. doi: 10.3389/fimmu.2024.1437869, 39351228 PMC11439652

[ref83] Zuccaro V. Petazzoni G. Mileto I. Corbella M. Asperges E. Sacchi P. . (2023). Gut microbiota and B cell receptor (BCR) inhibitors for the treatment of chronic lymphocytic leukemia: is biodiversity correlated with clinical response or immune-related adverse event occurrence? A cross-sectional study. Microorganisms 11:1305. doi: 10.3390/microorganisms11051305, 37317279 PMC10223513

