# Supplementary material for: Therapeutic ecology of the fiber-microbiota-barrier axis in leukemia: resilience, immune recovery and pharmacomicrobiomics
Source: Front Microbiol. 2026 Aug 25;17:1913406. doi: 10.3389/fmicb.2026.1913406 (PMC13552400; doi:10.3389/fmicb.2026.1913406)

Supplementary File S1. PubMed search strategy and screening flow

This supplementary file provides the complete PubMed search strategy and the PRISMA-style screening and public full-text retrieval flow. The complete editable evidence matrix is provided separately as Supplementary Table S1.

S1. PubMed Search Strategy

Database and dates

Database: PubMed.

Search date: 13 May 2026.

Publication date range: 13 May 2021 to 13 May 2026.

Full PubMed query

((leukemia[Title/Abstract] OR leukaemia[Title/Abstract] OR leukemic[Title/Abstract] OR "acute myeloid leukemia"[Title/Abstract] OR "acute lymphoblastic leukemia"[Title/Abstract] OR "acute lymphocytic leukemia"[Title/Abstract] OR "chronic lymphocytic leukemia"[Title/Abstract] OR "chronic myeloid leukemia"[Title/Abstract] OR AML[Title/Abstract] OR ALL[Title/Abstract] OR CLL[Title/Abstract] OR CML[Title/Abstract]) AND ("gut microbiota"[Title/Abstract] OR "gut microbiome"[Title/Abstract] OR "intestinal microbiota"[Title/Abstract] OR "intestinal microbiome"[Title/Abstract] OR "gut flora"[Title/Abstract] OR "intestinal flora"[Title/Abstract] OR dysbiosis[Title/Abstract] OR microbiota[Title] OR microbiome[Title]))

Screening and full-text retrieval

The search identified 276 PubMed records. After exclusion of 62 pediatric- or adolescent-focused records, 214 adult or non-pediatric records were screened for publicly accessible full text. Public full text was retrieved for 164 unique records, represented by 167 downloaded files. Following full-text relevance assessment, 137 records were retained in the evidence matrix (Supplementary Table S1).

S2. PRISMA-style Search Flow

Supplementary Figure S1. PubMed search and public full-text evidence-screening flow.

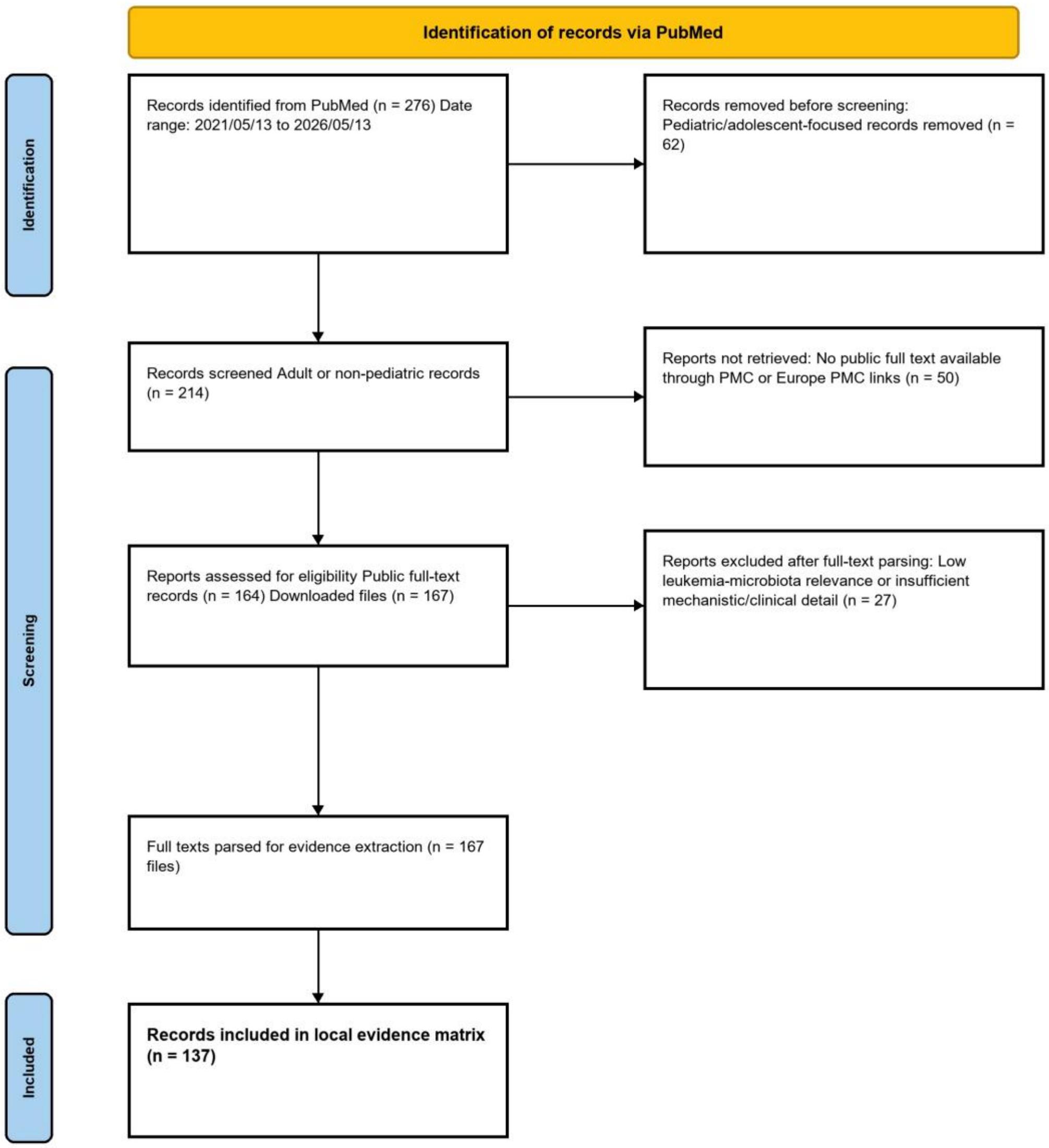

Supplement: Supplementary file 1 [file Data_Sheet_1.PDF]
